# Exploration of violet-to-blue thermally activated delayed fluorescence emitters based on “CH/N” and “H/CN” substitutions at diphenylsulphone acceptor. A DFT study

**DOI:** 10.3389/fchem.2023.1279355

**Published:** 2023-11-09

**Authors:** Aftab Hussain, Ahmad Irfan, Farah Kanwal, Muhammad Afzal, Aijaz Rasool Chaudhry, Mohamed Hussien, Muhammad Arif Ali

**Affiliations:** ^1^ School of Chemistry, University of the Punjab, Lahore, Pakistan; ^2^ Department of Chemistry, College of Science, King Khalid University, Abha, Saudi Arabia; ^3^ Department of Physics, College of Science, University of Bisha, Bisha, Saudi Arabia; ^4^ Institute of Chemistry, Baghdad Campus, The Islamia University of Bahawalpur, Bahawalpur, Pakistan

**Keywords:** diphenylsulphone (DPS), thermally activated delayed fluorescence (TADF), singlet-triplet energy gap (Δ*E*_ST_), organic light emitting diode (OLED), reverse intersystem crossing (RISC), DFT, TD-DFT, HOMO-LUMO

## Abstract

The violet-to-blue thermally activated delayed fluorescence (TADF) emitters were created employing several substituents based on 5,5-dimethyl-5,10-dihydropyrido [2,3-b][1,8] naphthyridine-diphenylsulphone (**DMDHPN-DPS**) called **1a** via “CH/N” and “H/CN” substitutions at the diphenylsulphone acceptor (**DPS**) moiety. The parent compound **1a** was selected from our former work after extensive research employing “CH/N” substitution on Dimethyl-acridine (**DMAC**) donor moiety. There is a little overlap amid the highest occupied molecular orbitals (HOMOs) and lowest un-occupied molecular orbitals (LUMOs) due to the distribution of HOMOs and LUMOs primarily on the **DMDHPN** donor and the **DPS** acceptor moieties, respectively. It resulted in a narrower energy gap (∆*E*_ST_) between the lowest singlet (S_1_) and triplet (T_1_) excited state. In nearly all derivatives, the steric hindrance results in a larger torsional angle (85°–98°) between the plane of the **DMDHPN** and the **DPS** moieties. The predicted Δ*E*_ST_ values of the compounds with “H/CN” substitution were lower than those of the comparable “CH/N” substituents, demonstrating the superiority of the reversible inter-system crossing (RISC) from the T_1_ → S_1_ state. All derivatives have emission wavelengths (*λ*_em_) in the range of 357–449 nm. The LUMO → HOMO transition energies in the S_1_ states are lowered by the presence of –CN groups or –N = atoms at the ortho or meta sites of a **DPS** acceptor unit, causing the *λ*_em_ values to red-shift. Furthermore, the *λ*_em_ showed a greater red-shift as there were more–CN groups or –N = atoms. Three of the derivatives named **1b**, **1g**, and **1h**, emit violet (394 nm, 399 nm, and 398 nm, respectively), while two others, **1f** and **1i**, emit blue shade (449 nm each) with reasonable emission intensity peak demonstrating that these derivatives are effective violet-to-blue TADF nominees. The lower Δ*E*_ST_ value for derivative **1i** (0.01 eV) with *λ*_em_ values of 449 nm make this molecule the finest choice for blue TADF emitter amongst all the studied derivatives. We believe our research might lead to the development of more proficient blue TADF-OLEDs in the future.

## 1 Introduction

Owing to their potential use as solid-state lightening sources and high-resolution flat panel display, organic light-emitting diodes (OLEDs) having multi-layer structures got a lot of interest since Tang and his colleagues initially described them (Tang and VanSlyke, 1987; Adachi et al., 2001; Zhang et al., 2014a; Chan et al., 2018; Liu et al., 2018; Wada et al., 2020). Because traditional fluorescent material can only harvest singlet excitons, OLEDs can only attain an internal quantum efficiency (IQE) of 25% (Shuai et al., 2000; Mehes et al., 2012; Sun et al., 2015). In contrast, noble heavy-metal-based phosphorescent materials may harvest both singlets as well as triplet excitons and hence can achieve an IQE of ≈ 100% through effective spin-orbit coupling interaction (SOC) (Adachi et al., 2001; Deaton et al., 2010; Zhang et al., 2012a; Xu et al., 2021). Noble metal-containing phosphorescent materials are, however, rather expensive and unreliable for blue emission. Therefore, a novel approach for obtaining high photoluminescence is required (Finkenzeller, 2008; Chi and Chou, 2010; Zhang et al., 2014a; Yu et al., 2022).

Adachi’s group, recently, has purported a novel triplet-harvesting mechanism named thermally activated delayed fluorescence (TADF) for highly proficient OLEDs as a workable alternative method with greater singlet yield (Endo et al., 2009; Mehes et al., 2012; Uoyama et al., 2012; Youn Lee et al., 2012; Li et al., 2020). For proficient TADF emitters, a narrower energy gap (∆*E*_ST_) amid the lowest singlet excited state (S_1_) and lowest triplet excited state (T_1_) is essential for reversible inter-system crossing (RISC) from the T_1_ → S_1_ state (Berberan-Santos and Garcia, 1996; Wada et al., 2020). The ∆*E*_ST_ is associated with exchange energy (***j***) between the HOMOs and LUMOs in a molecule (Lu et al., 2015a). A tiny spatial overlap (***ρ***) amid HOMO-LUMO is believed to be an essential component (Lu et al., 2015b) for obtaining a low ∆*E*_ST_ value, and this is realized by joining the donor (**D**) and the acceptor (**A**) fragments in structures via steric hindrance, for instance, bulk, spirojunction, or homoconjugation (Kawasumi et al., 2015; Lu et al., 2016; Liu et al., 2020; Zhang et al., 2022).

Diphenylsulphone (**DPS**) unit is a versatile fragment with favorable features for TADF materials owing to a twist angle in the center and a high electron-accepting capacity (Huang et al., 2014; Tao et al., 2014; Bryden and Zysman-Colman, 2021). The sulfonyl group has an electron-withdrawing characteristic because of significant electronegativity of the oxygen atom in the group. It can also prevent compounds from undergoing π-conjugation because of its tetrahedral structure. High-performance TADF-OLEDs have been recognized by several sulfone-based compounds. As a result, in recent years, **DPS** has emerged as the most popular electron-accepting moiety for TADF emitters (Ye et al., 2013; Wu et al., 2014; Li et al., 2015; Liu et al., 2015). Several **DPS**-containing compounds with TADF properties have previously been described by Adachi’s group (Zhang et al., 2012b; Ye et al., 2013; Wu et al., 2014; Li et al., 2015; Liu et al., 2015; Cui et al., 2020). Dimethylacridine-Diphenylsulphone (**DMAC-DPS**), among all the previously described **DPS**-based emitters, has been revealed to be a powerful blue TADF emitter with emission wavelength (*λ*_em_) of 460 nm in toluene and ∆*E*_ST_ (CT) value of 0.02 eV (Zhang et al., 2014a; Luo et al., 2016). In order to adjust the emission color, our group has published a chain of derivatives using H/R substitution on the D and A units (R = CH_3_ and CN) and “CH/N” substitution on the **D**-fragment (Wang et al., 2017a). It was discovered that modifying the **D** and **A** fragments with push-pull substituents is a useful technique for adjusting the *λ*_em_, reducing the ∆*E*_ST_, and enhancing the optical characteristics of designed molecules (Fan et al., 2016a; Lin et al., 2017a; Wang et al., 2017b; Yang et al., 2020; Jiao et al., 2021; Zhang et al., 2022). The “CH/N” as well as “H/CN” substitution on the acceptor unit, which results in bathochromically-shifted *λ*_em_ values and decreases ∆*E*_ST_ values, is a successful strategy, according to our analysis (Sun et al., 2008a; Sun et al., 2008b; Li et al., 2012).

**FIGURE 1:**
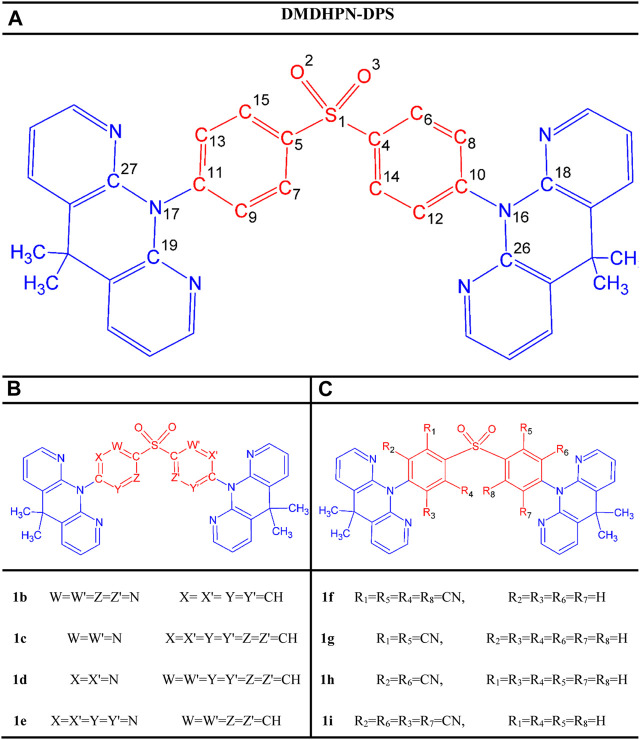
Scheme of the study showing the molecular structures of the parent molecule **(A)** and designed molecules **(B,C)** representing the CH/N and H/CN substituted derivatives, respectively.

In this contribution, we use the parent molecule **DMDHPN-DPS** (**1a**) as a starting point from our aforementioned report (Wang et al., 2017a). Then, by “CH/N” as well as “H/CN” substitution at meta and ortho sites of the **DPS** A-fragment, we were able to change the emission color to fashion blue TADF emitters. Figure 1 illustrates the structures of the parent molecule and all the symmetric substituted molecules. By computing ∆*E*_ST_ and *λ*_em_ values with the optimal Hartree-Fock percentage (OHF%) method in the exchange-correlation of the time-dependent density functional (TD-DFT) theory, we were able to analyse the TADF of these designed molecules.

## 2 Computational methodology

**FIGURE 2:**
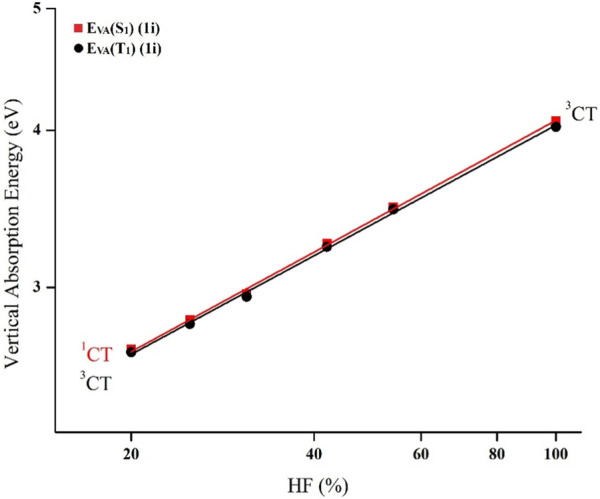
TD-DFT dependence of E_VA_ (S_1_) as well as E_VA_ (T_1_) on HF% in plotted on double log scale for the substituent **1i**.

At first, the Gaussian-09 program was utilized to perform the ground state geometry optimization (S_0_) for all of the derivatives employing B3LYP/6-31G(d) method. In addition, vibrational frequency analysis was accomplished to validate the local minima, and they turned up no imaginary frequencies (Irfan et al., 2014; Gao et al., 2017a; Gao et al., 2017b; Liu et al., 2022). Following that, the charge-transfer index (*q*), which is a measure of electron density re-distribution within a molecule, was determined from **D** to **A** using the HOMO and LUMO distribution. Subsequently, to examine the orbital composition using the Multiwfn tool, the optimal HF% (OHF%) was calculated using the relationship OHF = 42*q*. Based on S_0_ geometry, the vertical absorption energies for singlet *E*_VA_ (S_1_) as well as triplet *E*_VA_ (T_1_) were computed using different functionals including M06-HF, M06-2X, BMK, MPW1B95, PBE0, and B3LYP having different HF% of 100%, 54%, 42%, 31%, 25%, and 20%, respectively, with 6-31G(d) basis set. The HF percentage (HF%) of various functionals is given in Supplementary Figure S1. Afterward, the best-fit straight line of the double log plots of *E*_VA_ (S_1_, T_1_) against HF% was used to calculate the vertical excitation energy *E*_VA_ (S_1_, OHF) as seen in Figure 2 and S1. Eventually, the ∆*E*_ST_ and zero-zero transition energies (*E*_0-0_) were predicted using the following proven formulae of Adachi et al. (Huang et al., 2013; Zhang et al., 2014b; Tian et al., 2016; Cui et al., 2020).

$$E_{0-0}\left(1_{CT}\right)=E_{VA}\;\left(S_{1},OHF\right)-{∆E}_{V}-{∆E}_{stokes}$$ (1)

$$E_{0-0}\left(3_{CT}\right)=E_{0-0}\left(1_{CT}\right)-E_{VA}\;\left(S_{1},OHF\right)+\frac{E_{VA}\;\left(S_{1},OHF\right)}{E_{VA}\;\left(S_{1},B3LYP\right)}\times E_{VA}\;\left(T_{1},B3LYP\right)$$ (2)

$$E_{0-0}\left(3_{LE}\right)=E_{VA}\;\left(T_{1},B3LYP\right)/C-{∆E}_{stokes}$$ (3)

Here, ∆*E*_stokes_ (energy loss during Stokes-shift) is about 0.09 eV, and ∆*E*_V_ (difference in vibrational energy levels between the zero-zero transitions and the vertical transitions) is around 0.15 eV for the conjugated compounds. *E*_VA_ (T_1_, B3LYP) depicts the vertical excitation energy for the triplet (T_1_) state calculated via B3LYP/6-31G(d) method. The C is the correction factor whose value for BMK, M06-2X, and, M06-HF functionals is about 1.10, 1.18, and 1.30, respectively. The S_1_ state geometries were subsequently optimized using functionals with an HF% near the OHF (%). The MPW1B95 functional was selected for S_1_ state optimization for all the studied derivatives as the OHF was determined to be closest to 31% as shown in Table 1. Then, using the TD-MPW1B95/6-31G(d) method with a polarizable continuum model (PCM) in the toluene medium, the absorption (*λ*_ab_) and emission (*λ*_em_) wavelengths were determined based on optimized S_0_ and S_1_ state geometries respectively. The Gaussian-09 software is used for all calculations (Fan et al., 2016b; Cai et al., 2016; Liu et al., 2020). Software such as Multiwfn, PyMOlyze, Origin, Gaussview, and Gausssum were used for postprocessing the findings.

**TABLE 1:** Computed E_VA_ (S_1_), E_VA_ (T_1_), CT amount (q), OHF%, E_0-0_(^1^CT), E_0-0_(^3^CT), and E_0-0_ (^3^LE) employing several functionals and 6-31G(d) basis set based on B3LYP/6-31G(d) optimized geometries of the designed substituents **1f–1i**. **(1a–1e** **are shown in Supplementary Table S6)**.

Parameter	Functional	1f	1g		1h	1i
*E*_VA_ (S_1_) (eV)	B3LYP	2.8229	3.0381		3.0462	2.6772
	PBE1PBE	2.9791	3.2111		3.2030	2.8262
	MPWB95	3.1182	3.3666		3.3420	2.9645
	BMK	3.4277	3.7165		3.6559	3.2528
	M06-2X	3.6560	3.9666		3.8702	3.4756
	M06-HF	4.3066	4.7190		4.5220	4.0686
*E*_VA_ (T_1_) (eV)	B3LYP	2.6443	2.9874		3.0235	2.6639
	PBE1PBE	2.6950	3.0449		3.0970	2.8052
	MPWB95	2.8800	3.2675		3.3060	2.9488
	BMK	3.1009	3.4712		3.5358	3.2319
	M06-2X	3.3253	3.7124		3.7775	3.4619
	M06-HF	3.8135	4.0671		4.1056	4.0260
CT amount (*q*)		0.8417	0.8828		0.8539	0.8295
OHF%		35	37		36	35
*E*_*VA*_ (S_1_, OHF) (eV)		3.27	3.58		3.50	3.09
*E*_*0-0*_ (^1^CT) (eV)		3.03	3.34		3.26	2.85
*E*_*0-0*_ (^3^CT) (eV)		2.82	3.28		3.23	2.84
*E*_*0-0*_ (^3^LE) (eV)		2.77	3.06		3.07	2.90

## 3 Results and discussion

### 3.1 Optimized geometries at S_0_ and S_1_ states

Generally speaking, the photophysical characteristics of molecules with conjugated systems depend greatly on the dihedral angle and bond length. Stronger absorption, more effective emission, and improved fluorescence characteristics are frequently the results of optimal conjugation and planarity. Supplementary Table S1 shows the optimized geometrical parameters at S_0_ and S_1_ state of the parent and designed derivatives at B3LYP/6-31G(d) coupled with TD-MPW1B95/6-31G(d) level, employing DFT and TD-DFT, respectively. It is observed that the “CH/N” derivatives possess smaller C–N = bond lengths than the original C-C bond lengths at the pyrimidine/pyridine ring of the A-fragment because of the greater electronegativity of the nitrogen atom which draws the electron density towards itself and shortens the C–N = bond. In contrast, the C–C bond lengths in “H/CN” derivatives are longer than the prior C–C bond lengths because the –CN group will increase the electron-withdrawing strength of the A-moiety and elongates the C–C bond length inside the ring.

Bond lengths mostly alter on the substituent atom and nearby atom when compared to the original molecule **1a**. For instance, C_4_–N_14_ and C_4_–N_6_ bond lengths of **1b**, where C_14_ and C_6_ have been replaced by –N = atom, have decreased by 0.064 and 0.069 Å, respectively, in the S_0_ state and 0.071 and 0.070 Å in the S_1_ optimized state. Also, the lengths of the neighboring bonds C_12_–N_14_ and N_6_–C_8_ have lessened by 0.054 Å and 0.051 Å, respectively, in the S_0_ state and 0.072 Å in the S_1_ state. Conversely, for **1i**, where –CN group has been used to replace the H-atom at C_8_ and C_12_, and the C_8_–C_6_, C_10_–C_8_, C_12_–C_10_, and C_14_–C_12_ bond lengths have risen by 0.065, 0.011, 0.010 and, 0.066 Å in S_0_ state and 0.076, 0.004, 0.005, and 0.076 Å in S_1_ state, respectively. The bond length values for C_12_–C_14_ and C_6_–C_8_ are decreased by 0.019 Å in S_0_ to S_1_ transition while increased by 0.003 Å for C_8_–C_10_ and C_10_–C_12_. The geometrical parameters compared in the S_0_ state and S_1_ states for the parent and designed molecules showed a bond lengths alteration of up to 0.038 Å in C–C and C–N = bonds and up to 0.067 Å in C–S bonds. For the C–S bonds, the bond lengths alteration in the S_0_ state and S_1_ state are more pronounced. All of the proposed compounds have shorter C–S bonds in the S_1_ (0.045–0.067 Å) than they do in the S_0_. But their neighboring N–C and C–C bond lengths increase in the range of (0.002–0.023 Å) in S_1_ than those in the S_0_ state with only a few exceptions. Hence, it is obvious that the “CH/N” derivatives possess smaller C–N = bond lengths while “H/CN” derivatives possess larger C–C bond lengths. In both types of derivatives, “CH/N” and “H/CN”, the conjugation is increased compared with parent molecule **1a**, hence there is a red-shift in wavelength.

We know that the greater the value of the torsional/dihedral angle (*β*) between D and A fragments, the smaller will be the HOMO-LUMO overlap and the lower will be the value of △*E*_ST_. It can be seen from Supplementary Table S1 that all the derivatives have large dihedral angle values (∼85°–98°) between the D and A fragments in the S_0_ state except **1b** (70.2°) due to the greater steric hindrance and **1f** (115.8°) due to the smaller steric hindrance. The larger *β* values for almost all the derivatives are optimistic for blocking the electrical interaction between D and A units (Zhang et al., 2014b; Lin et al., 2017b).

### 3.2 Frontier molecular orbitals

**FIGURE 3:**
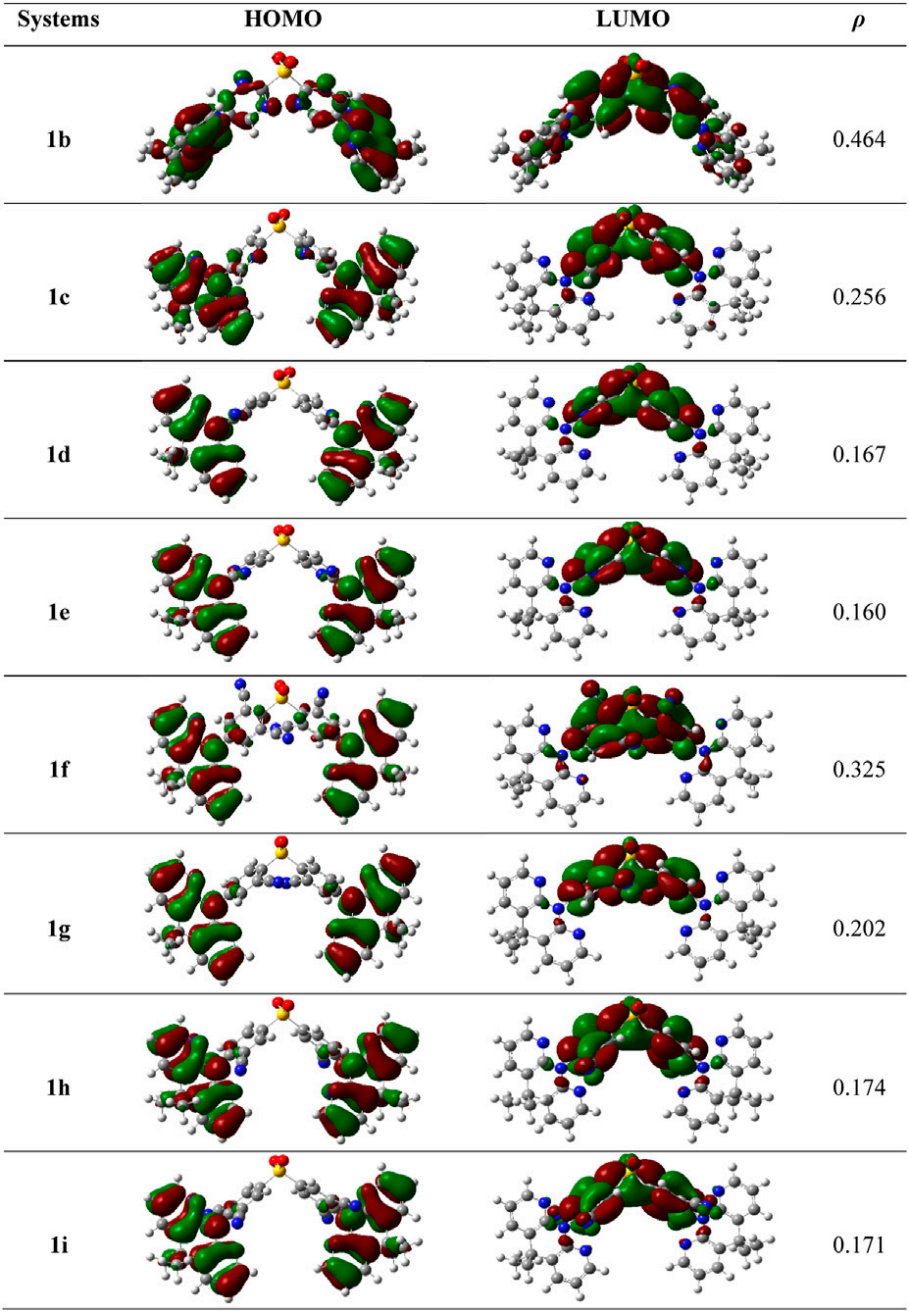
The electron density diagrams of HOMOs and LUMOs and the overlap between them in whole space for these studies compounds in S_0_ geometry. The isosurface value for FMOs is 0.02.

It is acknowledged that the frontier molecular orbitals (FMOs) offer crucial details on the nature of the TADF. The HOMO-LUMO electron density graphs at the S_0_ state are displayed in Figure 3. Using the below relation in the Multiwfn program, the overlap between HOMO and LUMO (*ρ*) was calculated using.

$$\int \left|\varphi_{i}\left(r\right)\right|\left|\varphi_{j}\left(r\right)\right|dr$$ (4)

**FIGURE 4:**
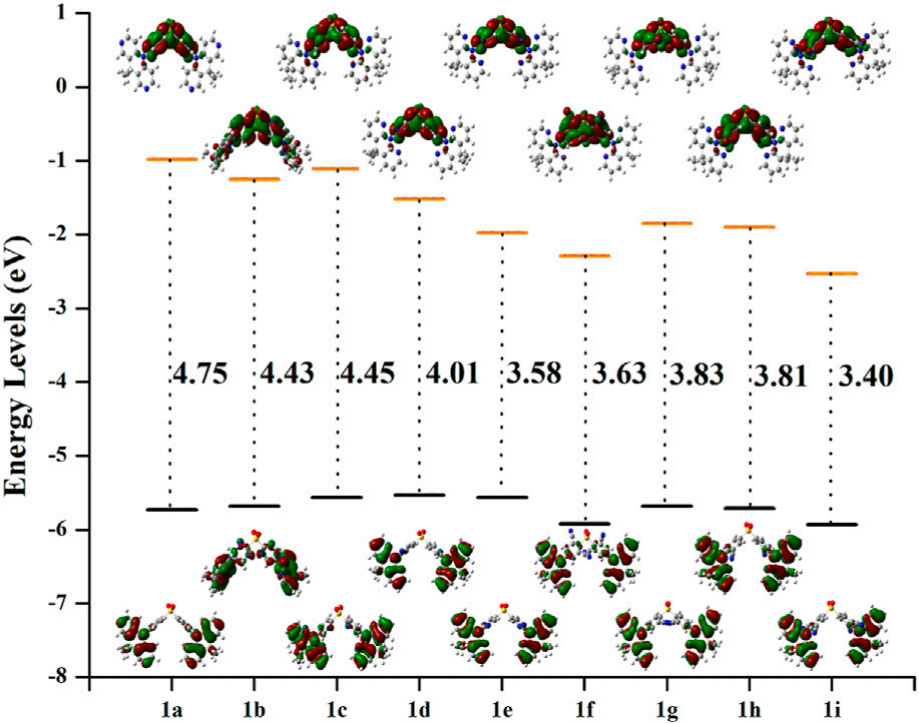
The electron density diagrams and HOMO-LUMO energetic gap of the studies molecules in the S_0_ state. The isosurface value for FMOs is 0.02.

As is obvious from Figure 3, the HOMOs are particularly localized on the **DMDHPN** donor fragment while the LUMOs are largely distributed on the **DPS** acceptor moiety. Additionally, we found that the *ρ* values are ranging from 0.160 to 0.256, showing an ease of charge transfer between HOMO and LUMO, and a low electron exchange energy (***j***), which makes the △*E*_ST_ very modest except **1f** (0.325) and **1b** (0.464). Because the LUMO is mainly found on the A-unit of substituted derivatives, more substantial changes can be found in LUMO energy levels than HOMO energy levels, as seen in Figure 4. The energetic gaps (Δ*E*_H-L_) between HOMO and LUMO are in the range of 3.84–4.69 eV in the S_1_ state and 3.40–4.45 eV in the S_0_ state.

When –N = atom or –CN groups are added to A-fragments, the values of Δ*E*_H-L_ are lower than they would be for the parent molecule because the enhanced electron-accepting capacity of the **DPS** acceptors lowers the LUMO energy level, as shown in Supplementary Table S2. However, the LUMO energy levels are reduced more effectively by the –CN group than by the –N = atom, which reduces the Δ*E*_H-L_.

Additionally, the shift in ∆*E*_H–L_ values is more apparent as the greater number of –CN groups or –N = atoms there are. Given that the *λ*_ab_ and *λ*_em_ values are connected to the ∆*E*_H–L_ values, (Li et al., 2012), the import of –CN groups or of –N = atoms to the A-fragments may alter the absorption and emission spectra, as illustrated in Supplementary Tables S4, S5. As a result, it is expected that the newly created derivatives would exhibit red-shifted *λ*_em_ when compared to the parent molecule **1a**.

### 3.3 Singlet-triplet energy gap

Using the Multiwfn program, the *E*_0-0_ (S_1_), *E*_0-0_ (^3^CT), as well as *E*_0-0_ (^3^LE) are attained based on the computed CT amount (*q*) (Table 1). At first, double log plots of *E*_VA_ (S_1_, T_1_) versus HF% for the compounds under investigation were plotted (Figure 2). Then the *E*_0-0_ (S_1_), *E*_0-0_ (^3^CT), as well as *E*_0-0_ (^3^LE) of these designed derivatives were computed using Eqs 1–3 as shown in Table 3. The calculated findings for the substituted molecules show that the addition of –CN groups or of –N = atoms on the A-moiety improves its capacity to pull electrons, which eventually leads to a decrease in the ∆*E*_ST_ value. Additionally, it is clear that as there are more –CN groups or of –N = atoms, the ∆*E*_ST_ values decrease, particularly at meta-position. It is also obvious that the “CH/N” derivatives have lower Δ*E*_ST_ values than the original molecule but are larger than the “H/CN” derivatives. When the lowest T_1_ state is taken into account, the computed *E*_0-0_ (^3^CT), as well as *E*_0-0_ (^3^LE), appear to have the lowest energy levels. The ^1^CT–^3^CT splitting is denoted by ∆*E*_ST_ (CT), while the energy difference amid the lowest T_1_ and the S_1_ state is denoted by Δ*E*_S1-T1_ (Huang et al., 2013). All the newly designed molecules are symmetric substitution derivatives and have lower Δ*E*_ST_ values as compared with a parent molecule. According to Table 2, for **1i,** the T_1_ state is ^3^CT in nature, which is ideal for an effectual RISC, whereas, for others, T_1_ states are ^3^LE in nature. The calculated ∆*E*_ST_ (CT) value using the OHF method for **1i** is 0.01 eV resulting from the small *ρ* value. The **1i** has the smallest ∆*E*_ST_ value compared with all the other substituted derivatives because 1) it is a “H/CN” substituted derivative which is more effective than “CH/N” substituted derivatives 2) two H-atoms have been replaced with two CN-groups which will further enhance the electron-withdrawing strength of the A-fragment 3) it is meta-substituted and is at a greater distance from sulphonyl group. Even though some other designed molecules, including the selected violet-blue to blue derivates like **1b**, **1f**, **1g**, and **1h**, possess a lower ^3^LE state, the effective TADF is made feasible via reversible internal conversion from ^3^LE to ^3^CT state followed by a subsequent RISC from ^3^CT to ^1^CT state. So, by enhancing the electron-withdrawing capacity of A, it is beneficial to raise the ^3^LE state and lower the ∆*E*_ST_ (Hussain et al., 2019; Hassan et al., 2022; Hussain et al., 2023).

**TABLE 2:** Computed ∆*E*_ST_ (CT) and Δ*E*_S1-T1_ for the studies molecules.

Molecules	*E*_0-0_ (^1^CT) (eV)	*E*_0-0_ (^3^CT) (eV)	*E*_0-0_ (^3^LE) (eV)	∆*E*_ST_ (CT) (eV)	∆*E*_S1-T1_ (eV)
**1a**	4.04	3.46	3.07 (T_1_)	0.58	0.97
**1b**	3.44	3.21	3.05 (T_1_)	0.23	0.39
**1c**	3.75	3.46	3.03 (T_1_)	0.29	0.72
**1d**	3.54	3.52	3.10 (T_1_)	0.02	0.44
**1e**	3.14	3.13	3.04 (T_1_)	0.01	0.10
**1f**	3.03	2.82	2.77 (T_1_)	0.21	0.26
**1g**	3.34	3.27	3.07 (T_1_)	0.07	0.27
**1h**	3.26	3.23	3.07 (T_1_)	0.03	0.19
**1i**	2.85	2.84 (T_1_)	2.90	0.01	−0.05

### 3.4 Photophysical properties

**FIGURE 5:**
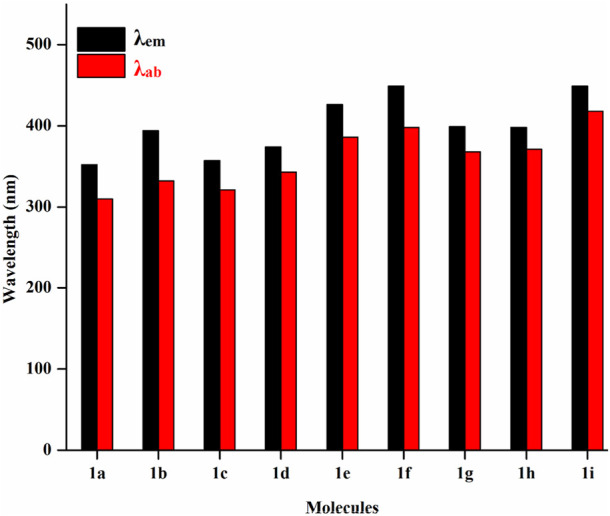
Absorption and emission spectrum of the studied molecules.

Figure 5 displays the emission and absorption spectra of the parent molecule and all derivatives. Table 3 lists the estimated emission and absorption wavelengths (*λ*_em_ and *λ*_ab_) in the toluene medium at the TD-MPW1B95/6-31G(d) theory level. All the investigation molecules have *λ*_em_ and *λ*_ab_ values corresponding to the values of Δ*E*_H-L_. The designed derivatives show that the *λ*_em_ and *λ*_ab_ values range from 357 to 449 nm and 321–418 nm, respectively. The *λ*_em_ bands instigate from S_1_ → S_0_ transitions which is mainly the transference of the electrons from LUMO → HOMO, and the electronic shifts are π* → π type. The addition of –CN group or –N = atom to **DPS** acceptor moiety has resulted in the bathochromically-shifted values for *λ*_em_ and *λ*_ab_. Furthermore, by increasing the numbers of –CN groups or –N = atoms, the *λ*_em_, and *λ*_ab_ values display a more significant red-shift. Particularly, the derivatives **1f** and **1i,** display a more noticeable red-shift in *λ*_em_ of about 97 nm each. Additionally, the meta-positioned derivatives show more red-shift in *λ*_em_ and *λ*_ab_ values than ortho-positions with the same number of –CN groups or of –N = atoms. Actually, at meta-position, the –CN group or –N = atom is at a larger distance from the sulphonyl group and experiences less steric hindrance compared to the ortho position which experiences more steric repulsion. It will increase the stability and charge transfer characters of the substituted derivatives which results in lowering the Δ*E*_ST_ and increasing the *λ*_em_ value. It is evident from the calculated results that the three molecules named **1b**, **1g**, and **1h** are showing violet-blue emission (394, 399, and 398 nm) and two of them named **1f** and **1i** are showing blue emission of 449 nm with reasonable emission intensity peak demonstrating that the studied compounds are effective violet-blue to blue TADF materials. We can see that both **1f** and **1i** have the same value of emission wavelength (*λ*_em_) of 449 nm lying in the pure blue region but **1i** is regarded as a comparatively better TADF candidate as it has a lower ΔE_ST_ 0.01 eV value as compared to **1f** (0.26 eV). The constructed molecules demonstrated that the sequence of *λ*_ab_, as well as *λ*_em_, is coherent with the propensity of the electron-withdrawing strength of A-fragment.

**TABLE 3:** Calculated λ_ab_ and λ_em_ values in toluene for the studied molecules employing TD-MPW1B95/6-31G(d) method based on S_0_ and S_1_ state optimized geometries, respectively, along with Stoke Shift (∆υ) values.

Molecule	Wavelength *λ*_ab_ (nm)	Oscillator strength (*f*)	Wavelength *λ*_em_ (nm)	Oscillator strength (*f*)	Stoke shift (∆υ) (∆υ = *λ*_em_−*λ*_ab_)
**1a**	310	0.0015	352	0.1385	42
**1b**	332	0.0723	394	0.1682	62
**1c**	321	0.0069	357	0.2464	36
**1d**	343	0.0010	374	0.0005	31
**1e**	386	0.0003	426	0.0001	40
**1f**	398	0.1921	449	0.0081	51
**1g**	368	0.0182	399	0.0473	31
**1h**	371	0.0047	398	0.0159	27
**1i**	418	0.0005	449	0.0165	31

The energy (or wavelength) differential between a molecule’s absorption (*λ*_ab_) and emission (*λ*_em_) maxima is called as the Stokes-shift (**∆υ**). It represents the energy loss occurring during the S_1_ → S_0_ transition. In general, a smaller Stokes-shift is advantageous since it decreases the spectral overlap between excitation and emission signals. Since self-absorption is reduced by a narrow Stokes-shift, more of the absorbed energy is transformed into light output, enhancing OLEDs’ total energy efficiency. Every constructed molecule, except **1b** and **1f**, exhibits a smaller stokes-shift than the original molecule as shown in Table 3. It is also obvious that the “H/CN” substituents exhibit a relatively smaller value compared with “CH/N” derivatives hence more effective in reducing the energy loss during the S_1_ → S_0_ transition.

## 4 Conclusion

In conclusion, we have projected the photophysical as well as electronic characteristics for a variety of freshly created substituents to improve the efficacies for blue TADF emitters based on the parent system **DMDHPN-DPS**. The HOMOs are primarily concentrated on the **DMDHPN**-donor moiety, whereas the LUMOs are located at the **DPS**-acceptor unit. The *λ*_em_ of all the designed derivatives ranges from 357 to 449 nm. The import of –CN groups or of –N = atoms on **DPS** moiety lowers the transition energy from LUMO to HOMO, causing a red-shift in *λ*_em_. Additionally, the red-shift in *λ*_em_ values becomes more significant by increasing the quantity of –CN groups or of –N = atoms. The derivatives **1f** and **1i** display a more evident red-shift of 97 nm. Additionally, meta-position substituents exhibit greater red-shifted *λ*_em_ values than ortho-position substituents with the same number of –CN groups or of –N = atoms. Actually, at meta-position, the –CN group or –N = atom is at a larger distance from the sulphonyl group and experiences less steric hindrance compared to the ortho position which experiences more steric repulsion. It will increase the stability and charge transfer characters of the substituted derivatives which result in lowering the Δ*E*_ST_ and increasing the *λ*_em_ value. The small *ρ* values for the designed molecules due to the greater *β* values of 85°–98° contribute to the realization of the charge transfer state and small Δ*E*_ST_. The estimated findings showed that, out of all the analyzed molecules, three violet-blue emitters (394–399 nm) and two blue emitters (449 nm) with reasonable emission intensity and smaller Δ*E*_ST_ are favorable to be effective violet-blue to blue TADF emitters with **1i** being the best TADF candidate as it has smallest Δ*E*_ST_ values of 0.01 eV. We believe that in the future, understanding and building effective violet-blue to blue TADF-based OLEDs will be made easier with the aid of our theoretical designs.

## Data Availability

The original contributions presented in the study are included in the article/Supplementary Material, further inquiries can be directed to the corresponding authors.
