# Supplementary material for: Exploration of violet-to-blue thermally activated delayed fluorescence emitters based on “CH/N” and “H/CN” substitutions at diphenylsulphone acceptor. A DFT study
Source: Front Chem. 2023 Nov 9;11:1279355. doi: 10.3389/fchem.2023.1279355 (PMC10666053; doi:10.3389/fchem.2023.1279355)
Supplement: Supplementary file 1 [file DataSheet1.docx]

**Supplementary Information**

**Exploration of Violet-to-Blue Thermally Activated Delayed Fluorescence Emitters Based on "CH/N" and "H/CN" Substitutions at Diphenylsulphone Acceptor. A DFT study**

Aftab Hussain^a^*, Ahmad Irfan^b^, Farah Kanwal^a^*, Muhammad Afzal^a^, Mohamed Hussien^b^, Aijaz Rasool Chaudhry^c^,

*^a^School of Chemistry, University of the Punjab, Lahore 54590, Pakistan*

*^b^Department of Chemistry, College of Science, King Khalid University, Abha 61413, P.O. Box 9004, Saudi Arabia*

*^c^Department of Physics, College of Science, University of Bisha, Bisha 61922, P.O. Box 551, Saudi Arabia*

* Corresponding author. Tel.: +923426224761 (AH)

E-mail address: [aftab.chem@pu.edu.pk](mailto:aftab.chem@pu.edu.pk); [farahkchem@yahoo.com](mailto:farahkchem@yahoo.com)

**Table of Contents**

[**Figure S1** The HF percentage (HF%) of various functionals. 2](#_Toc142506745)

[**Table S1.** Calculated structural parameters like bond lengths, bond angles and dihedral angles in the S_0_ and S_1_ optimized geometries using B3LYP/6-31g(d) and MPW1B95/6-31g(d) methods, respectively. 2](#_Toc142506746)

[**Table S2.** HOMO-LUMO Energy gap in the S_0_ and S_1_ state optimized geometries using B3LYP/6-31g(d) and MPW1B95/6-31g(d) methods, respectively. 7](#_Toc142506747)

[**Table S3.** Calculated Overlap (*ρ*) using Multiwfn program and ∆*E*_ST_ (in eV) values using OHF% Method. 7](#_Toc142506748)

[**Table S4.** Calculated emission wavelength (*λ*_em)_ values and main conﬁguration for the investigated molecules using TD-MPW1B95/6-31g(d) method based on optimized S_1_ geometry in Toluene media using PCM Model_._ 8](#_Toc142506749)

[**Table S5.** Calculated absorption wavelength (*λ*_ab_) values and main conﬁguration for investigated molecules using TD-MPW1B95/6-31g(d) method based on optimized S_0_ geometry in Toluene media using PCM Model_._ 9](#_Toc142506750)

[**Table S6.** Calculated *E*_VA_(S_1_) and *E*_VA_(T_1_) using various exchange-correlation functionals and 6-31G* basis set based on B3LYP optimized geometries, and calculated CT amount (*q*), optimal HF% (OHF), *E*_0-0_(^3^LE), *E*_0-0_(^1^CT) and *E*_0-0_(^3^CT) of investigated molecules. 10](#_Toc142506751)

[**Figure S2** Dependence of *E*_VA_(S_1_) and *E*_VA_(T_1_) on the HF% in TD-DFT plotted on a log-log scale. 12](#_Toc142506752)





# **Figure S1** The HF percentage (HF%) of various functionals.

# **Table S1.** Calculated structural parameters like bond lengths, bond angles and dihedral angles in the S_0_ and S_1_ optimized geometries using B3LYP/6-31g(d) and MPW1B95/6-31g(d) methods, respectively.

| 1a | S_0_ | S_1_ | Diff. |
| --- | --- | --- | --- |
| R(1,2) | 1.464 | 1.471 | 0.007 |
| R(1,4) | 1.785 | 1.753 | 0.032 |
| R(4,6) | 1.392 | 1.412 | 0.020 |
| R(4,14) | 1.392 | 1.412 | 0.020 |
| R(6,8) | 1.388 | 1.379 | 0.009 |
| R(8,10) | 1.392 | 1.404 | 0.012 |
| R(10,12) | 1.392 | 1.404 | 0.012 |
| R(12,14) | 1.389 | 1.378 | 0.011 |
| R(10,16) | 1.433 | 1.436 | 0.003 |
| R(16,18) | 1.393 | 1.392 | 0.001 |
| R(16,26) | 1.392 | 1.390 | 0.002 |
| *α*(2,1,3) | 121.8 | 121.4 | 0.4 |
| *α*(4,1,5) | 104.5 | 105.8 | 1.3 |
| *α*(1,4,6) | 119.3 | 119.9 | 0.6 |
| *α*(8,10,16) | 119.7 | 119.9 | 0.2 |
| *α*(10,16,18) | 119.3 | 119.7 | 0.4 |
| *β*(8,10,16,26) | 96.5 | 111.1 | 14.6 |

| 1b | S_0_ | S_1_ | Diff. |
| --- | --- | --- | --- |
| R(1,2) | 1.466 | 1.453 | 0.013 |
| R(1,4) | 1.837 | 1.770 | 0.067 |
| R(4,6) | 1.323 | 1.342 | 0.019 |
| R(4,14) | 1.328 | 1.341 | 0.013 |
| R(6,8) | 1.337 | 1.307 | 0.030 |
| R(8,10) | 1.394 | 1.404 | 0.010 |
| R(10,12) | 1.396 | 1.404 | 0.008 |
| R(12,14) | 1.335 | 1.306 | 0.029 |
| R(10,16) | 1.429 | 1.409 | 0.020 |
| R(16,18) | 1.406 | 1.395 | 0.011 |
| R (16,26) | 1.405 | 1.393 | 0.012 |
| *α*(2,1,3) | 121.2 | 122.1 | 0.9 |
| *α*(4,1,5) | 101.5 | 102.2 | 0.7 |
| *α*(1,4,6) | 116.2 | 117.4 | 1.2 |
| *α*(8,10,16) | 121.6 | 121.9 | 0.0 |
| *α*(10,16,18) | 119.3 | 119.7 | 0.6 |
| *β*(8,10,16,26) | 70.2 | 62.82 | 7.4 |

| 1c | S_0_ | S_1_ | Diff. |
| --- | --- | --- | --- |
| R(1,2) | 1.472 | 1.459 | 0.013 |
| R(1,4) | 1.820 | 1.762 | 0.058 |
| R(4,6) | 1.391 | 1.403 | 0.012 |
| R(4,14) | 1.328 | 1.342 | 0.014 |
| R(6,8) | 1.394 | 1.372 | 0.022 |
| R(8,10) | 1.392 | 1.405 | 0.013 |
| R(10,12) | 1.398 | 1.401 | 0.003 |
| R(12,14) | 1.333 | 1.311 | 0.022 |
| R(10,16) | 1.437 | 1.418 | 0.019 |
| R(16,18) | 1.402 | 1.393 | 0.009 |
| R(16,26) | 1.401 | 1.392 | 0.009 |
| *α*(2,1,3) | 120.3 | 121.5 | 1.2 |
| *α*(4,1,5) | 103.2 | 102.95 | 0.22 |
| *α*(1,4,6) | 119.1 | 119.1 | 0.00 |
| *α*(8,10,16) | 121.2 | 121.2 | 0.00 |
| *α*(10,16,18) | 119.2 | 119.9 | 0.7 |
| *β*(8,10,16,26) | 85.0 | 60.9 | 24.1 |

| 1d | S_0_ | S_1_ | Diff. |
| --- | --- | --- | --- |
| R(1,2) | 1.471 | 1.464 | 0.007 |
| R(1,4) | 1.799 | 1.741 | 0.058 |
| R(4,6) | 1.396 | 1.410 | 0.014 |
| R(4,14) | 1.396 | 1.406 | 0.010 |
| R(6,8) | 1.389 | 1.374 | 0.015 |
| R(8,10) | 1.397 | 1.392 | 0.005 |
| R(10,12) | 1.331 | 1.335 | 0.004 |
| R(12,14) | 1.336 | 1.317 | 0.019 |
| R(10,16) | 1.440 | 1.442 | 0.002 |
| R(16,18) | 1.399 | 1.382 | 0.017 |
| R(16,26) | 1.398 | 1.379 | 0.019 |
| *α*(2,1,3) | 122.1 | 121.8 | 0.3 |
| *α*(4,1,5) | 104.9 | 106.8 | 1.9 |
| *α*(1,4,6) | 120.3 | 121.0 | 0.7 |
| *α*(8,10,16) | 119.6 | 120.2 | 0.6 |
| *α*(10,16,18) | 118.9 | 119.2 | 0.3 |
| *β*(8,10,16,26) | 96.6 | 95.4 | 1.2 |

| 1e | S_0_ | S_1_ | Diff. |
| --- | --- | --- | --- |
| R(1,2) | 1.469 | 1.463 | 0.006 |
| R(1,4) | 1.795 | 1.737 | 0.058 |
| R(4,6) | 1.395 | 1.408 | 0.013 |
| R(4,14) | 1.396 | 1.408 | 0.012 |
| R(6,8) | 1.334 | 1.317 | 0.017 |
| R(8,10) | 1.334 | 1.329 | 0.005 |
| R(10,12) | 1.335 | 1.329 | 0.006 |
| R(12,14) | 1.333 | 1.317 | 0.016 |
| R(10,16) | 1.432 | 1.442 | 0.010 |
| R(16,18) | 1.395 | 1.378 | 0.017 |
| R(16,26) | 1.394 | 1.376 | 0.018 |
| *α*(2,1,3) | 122.5 | 122.3 | 0.2 |
| *α*(4,1,5) | 104.95 | 106.9 | 2 |
| *α*(1,4,6) | 121.1 | 121.7 | 0.6 |
| *α*(8,10,16) | 116.2 | 115.4 | 0.8 |
| *α*(10,16,18) | 118.8 | 118.9 | 0.1 |
| *β*(8,10,16,26) | 88.2 | 90.9 | 2.7 |

| 1f | S_0_ | S_1_ | Diff. |
| --- | --- | --- | --- |
| R(1,2) | 1.463 | 1.455 | 0.008 |
| R(1,4) | 1.822 | 1.758 | 0.064 |
| R(4,6) | 1.407 | 1.418 | 0.011 |
| R(4,14) | 1.410 | 1.423 | 0.023 |
| R(6,8) | 1.405 | 1.387 | 0.018 |
| R(8,10) | 1.391 | 1.390 | 0.001 |
| R(10,12) | 1.393 | 1.386 | 0.007 |
| R(12,14) | 1.401 | 1.385 | 0.016 |
| R(10,16) | 1.430 | 1.434 | 0.004 |
| R(16,18) | 1.409 | 1.386 | 0.023 |
| R(16,26) | 1.408 | 1.384 | 0.024 |
| *α*(2,1,3) | 121.6 | 121.4 | 0.2 |
| *α*(4,1,5) | 105.3 | 106.8 | 1.5 |
| *α*(1,4,6) | 120.7 | 121.7 | 1.0 |
| *α*(8,10,16) | 120.3 | 119.8 | 0.5 |
| *α*(10,16,18) | 119.5 | 119.3 | 0.2 |
| *β*(8,10,16,26) | 115.8 | 84.4 | 31.4 |

| 1g | S_0_ | S_1_ | Diff. |
| --- | --- | --- | --- |
| R(1,2) | 1.468 | 1.459 | 0.009 |
| R(1,4) | 1.811 | 1.754 | 0.067 |
| R(4,6) | 1.392 | 1.397 | 0.005 |
| R(4,14) | 1.411 | 1.426 | 0.015 |
| R(6,8) | 1.395 | 1.376 | 0.019 |
| R(8,10) | 1.393 | 1.405 | 0.012 |
| R(10,12) | 1.394 | 1.376 | 0.018 |
| R(12,14) | 1.401 | 1.396 | 0.005 |
| R(10,16) | 1.438 | 1.437 | 0.001 |
| R(16,18) | 1.402 | 1.386 | 0.016 |
| R(16,26) | 1.402 | 1.385 | 0.017 |
| *α*(2,1,3) | 121.1 | 120.7 | 0.4 |
| *α*(4,1,5) | 104.93 | 104.98 | 0.05 |
| *α*(1,4,6) | 117.3 | 117.4 | 0.1 |
| *α*(8,10,16) | 120.3 | 119.4 | 0.9 |
| *α*(10,16,18) | 119.2 | 119.8 | 0.6 |
| *β*(8,10,16,26) | 97.9 | 74.6 | 23.3 |

| 1h | S_0_ | S_1_ | Diff. |
| --- | --- | --- | --- |
| R(1,2) | 1.470 | 1.461 | 0.009 |
| R(1,4) | 1.805 | 1.760 | 0.045 |
| R(4,6) | 1.396 | 1.411 | 0.015 |
| R(4,14) | 1.392 | 1.384 | 0.008 |
| R(6,8) | 1.393 | 1.381 | 0.012 |
| R(8,10) | 1.392 | 1.382 | 0.010 |
| R(10,12) | 1.409 | 1.423 | 0.014 |
| R(12,14) | 1.401 | 1.392 | 0.009 |
| R(10,16) | 1.433 | 1.429 | 0.004 |
| R(16,18) | 1.403 | 1.386 | 0.017 |
| R(16,26) | 1.402 | 1.385 | 0.017 |
| *α*(2,1,3) | 122.2 | 121.9 | 0.3 |
| *α*(4,1,5) | 104.5 | 105.1 | 0.6 |
| *α*(1,4,6) | 119.3 | 119.8 | 0.5 |
| *α*(8,10,16) | 120.8 | 122.2 | 1.4 |
| *α*(10,16,18) | 119.2 | 118.7 | 0.5 |
| *β*(8,10,16,26) | 86.3 | 87.2 | 0.9 |

| 1i | S_0_ | S_1_ | Diff. |
| --- | --- | --- | --- |
| R(1,2) | 1.468 | 1.460 | 0.008 |
| R(1,4) | 1.807 | 1.754 | 0.053 |
| R(4,6) | 1.392 | 1.400 | 0.008 |
| R(4,14) | 1.392 | 1.400 | 0.008 |
| R(6,8) | 1.402 | 1.383 | 0.019 |
| R(8,10) | 1.405 | 1.408 | 0.003 |
| R(10,12) | 1.406 | 1.409 | 0.003 |
| R(12,14) | 1.401 | 1.382 | 0.019 |
| R(10,16) | 1.425 | 1.422 | 0.003 |
| R(16,18) | 1.405 | 1.387 | 0.018 |
| R(16,26) | 1.404 | 1.386 | 0.018 |
| *α*(2,1,3) | 122.8 | 122.8 | 0.0 |
| *α*(4,1,5) | 104.6 | 105.4 | 0.8 |
| *α*(1,4,6) | 119.0 | 119.5 | 0.5 |
| *α*(8,10,16) | 120.4 | 120.2 | 0.2 |
| *α*(10,16,18) | 119.0 | 119.2 | 0.2 |
| *β*(8,10,16,26) | 87.3 | 82.5 | 4.8 |

# **Table S2.** HOMO-LUMO Energy gap in the S_0_ and S_1_ state optimized geometries using B3LYP/6-31g(d) and MPW1B95/6-31g(d) methods, respectively.

| **System** | **S_0_ geometry** | | | **S_1_ geometry** | | |
| --- | --- | --- | --- | --- | --- | --- |
|  | **E_HOMO_** | **E_LUMO_** | **E_L-H_** | **E_LUMO_** | **E_HOMO_** | **E_L-H_** |
| **1a** | -5.73 | -0.98 | 4.75 | 5.68 | 1.25 | 4.43 |
| **1b** | -5.68 | -1.25 | 4.43 | -6.0 | -1.56 | 4.44 |
| **1c** | -5.56 | -1.11 | 4.45 | -5.85 | -1.16 | 4.69 |
| **1d** | -5.53 | -1.52 | 4.01 | -5.86 | -1.47 | 4.39 |
| **1e** | -5.56 | -1.98 | 3.58 | -5.88 | -1.92 | 3.96 |
| **1f** | -5.92 | -2.29 | 3.63 | -6.22 | -2.31 | 3.91 |
| **1g** | -5.68 | -1.85 | 3.83 | -6.01 | -1.79 | 4.22 |
| **1h** | -5.71 | -1.90 | 3.81 | -6.01 | -1.76 | 4.25 |
| **1i** | -5.93 | -2.53 | 3.40 | -6.24 | -2.40 | 3.84 |

# **Table S3.** Calculated Overlap (*ρ*) using Multiwfn program and ∆*E*_ST_ (in eV) values using OHF% Method.

| **System** | **Overlap** (*ρ*) | **∆*E*_ST_** |
| --- | --- | --- |
| **1a** | 0.225 | 0.97 |
| **1b** | 0.464 | 0.39 |
| **1c** | 0.256 | 0.72 |
| **1d** | 0.167 | 0.44 |
| **1e** | 0.160 | 0.10 |
| **1f** | 0.325 | 0.26 |
| **1g** | 0.202 | 0.27 |
| **1h** | 0.174 | 0.19 |
| **1i** | 0.171 | 0.01 |

# **Table S4.** Calculated emission wavelength (*λ*_em)_ values and main conﬁguration for the investigated molecules using TD-MPW1B95/6-31g(d) method based on optimized S_1_ geometry in Toluene media using PCM Model_._

| **System** | **Excited State** | **Main Configuration/ Transition (%T=T*100)** | **Wavelength *λ*_em_ (nm)** | ***E*_S_@S_0_**  **(eV)** | **Oscillator Strength (*f*)** |
| --- | --- | --- | --- | --- | --- |
| **1a** | S_1_ | LUMO → HOMO (94) | 352 | 3.52 | 0.1385 |
| ***f*_max_** | S_11_ | LUMO → HOMO (84) | 266 | 4.66 | 0.4215 |
| **1b** | S_1_ | LUMO → HOMO (86) | 394 | 3.15 | 0.1682 |
| ***f*_max_** | S_9_ | L+7→H (51), L+6→H-1 (46) | 277 | 4.48 | 0.3671 |
| **1c** | S_1_ | LUMO → HOMO (88) | 357 | 3.48 | 0.2464 |
| ***f*_max_** | S_15_ | LUMO→H-2 (66) | 256 | 4.85 | 0.3278 |
| **1d** | S_1_ | LUMO → HOMO (90) | 374 | 3.31 | 0.0005 |
| ***f*_max_** | S_14_ | LUMO → H-2 (65) | 263 | 4.72 | 0.4237 |
| **1e** | S_1_ | LUMO → HOMO (91) | 426 | 2.91 | 0.0000 |
| ***f*_max_** | S_14_ | L+7→H (47), L+6→H-1 (47) | 277 | 4.48 | 0.3962 |
| **1f** | S_1_ | LUMO → HOMO (83) | 449 | 2.76 | 0.0081 |
| ***f*_max_** | S_9_ | LUMO → H-2 (89) | 291 | 4.26 | 0.3223 |
| **1g** | S_1_ | LUMO → HOMO (86) | 399 | 3.11 | 0.0473 |
| ***f*_max_** | S_11_ | L→H-2 (25), L+7→H-1 (35) | 277 | 4.48 | .06825 |
| **1h** | S_1_ | LUMO → HOMO (74) | 398 | 3.12 | 0.0159 |
| ***f*_max_** | S_10_ | L+6→H-1 (43), L+7→H (43) | 274 | 4.52 | 0.3700 |
| **1i** | S_1_ | LUMO → HOMO (84) | 449 | 2.76 | 0.0165 |
| ***f*_max_** | S_20_ | L+6→H-1 (36), L→H-7 (21) | 270 | 4.60 | 0.3179 |

# **Table S5.** Calculated absorption wavelength (*λ*_ab_) values and main conﬁguration for investigated molecules using TD-MPW1B95/6-31g(d) method based on optimized S_0_ geometry in Toluene media using PCM Model_._

| **System** | **Excited State** | **Main Configuration/ Transition (%T=T*100)** | **Wavelength *λ*_ab_ (nm)** | ***E*_S_@S_0_**  **(eV)** | **Oscillator Strength (*f*)** |
| --- | --- | --- | --- | --- | --- |
| **1a** | S_1_ | H-1→LUMO (91%) | 310 | 3.9951 | 0.0015 |
| ***f* _max_** | S_8_ | H-1→L+5 (47%), HOMO→L+4 (48%) | 276 | 4.4855 | 0.3648 |
| **1b** | S_1_ | H-1→LUMO (53%) HOMO→L+2 (38%) | 332 | 3.7296 | 0.0723 |
| ***f* _max_** | S_10_ | HOMO→L+7 (49%)  H-1→L+6 (47%) | 276 | 4.4925 | 0.6394 |
| **1c** | S_1_ | HOMO→LUMO (65%) H-1→L+1 (30%) | 321 | 3.8595 | 0.0069 |
| ***f* _max_** | S_7_ | HOMO→L+6 (48%)  H-1→L+7 (48%) | 277 | 4.4821 | 0.4733 |
| **1d** | S_1_ | HOMO→LUMO (85%) H-1→L+1 (7%) | 343 | 3.6112 | 0.0010 |
| ***f* _max_** | S_17_ | H-2→LUMO (88%)  H-3→L+1 (4%) | 248 | 4.9968 | 0.6192 |
| **1e** | S_1_ | HOMO→LUMO (86%) H-1→L+3 (12%) | 386 | 3.2122 | 0.0003 |
| ***f* _max_** | S_16_ | H-1→L+7 (42%) HOMO→L+6 (42%) | 274 | 4.5217 | 0.4852 |
| **1f** | S_1_ | HOMO→LUMO (75%) H-1→L+1 (22%) | 398 | 3.1182 | 0.1921 |
| ***f* _max_** | S_12_ | HOMO→L+7 (40%)  H-1→L+6 (38%) | 274 | 4.5234 | 0.3814 |
| **1g** | S_1_ | HOMO→LUMO (74%) H-1→L+1 (24%) | 368 | 3.3666 | 0.0182 |
| ***f* _max_** | S_10_ | H-1→L+6 (48%) HOMO→L+7 (49%) | 275 | 4.5056 | 0.4910 |
| **1h** | S_1_ | H-1→LUMO (73%) HOMO→L+1 (22%) | 371 | 3.3420 | 0.0047 |
| ***f* _max_** | S_10_ | H-1→L+7 (48%) HOMO→L+6 (48%) | 273 | 4.5360 | 0.4547 |
| **1i** | S_1_ | HOMO→LUMO (78%) H-1→L+3 (19%) | 418 | 2.9645 | 0.0005 |
| ***f* _max_** | S_15_ | H-1→L+6 (44%) HOMO→L+7 (45%) | 270 | 4.5866 | 0.4094 |

# **Table S6.** Calculated *E*_VA_(S_1_) and *E*_VA_(T_1_) using various exchange-correlation functionals and 6-31G* basis set based on B3LYP optimized geometries, and calculated CT amount (*q*), optimal HF% (OHF), *E*_0-0_(^3^LE), *E*_0-0_(^1^CT) and *E*_0-0_(^3^CT) of investigated molecules.

| **Parameter** | **Functional** | **1a** | **1b** | **1c** | **1d** | **1e** |
| --- | --- | --- | --- | --- | --- | --- |
| ***E*_VA_ (S_1_)**  **(eV)** | B3LYP | 3.8220 | 3.4716 | 3.5665 | 3.2824 | 2.8739 |
|  | PBE1PBE | 3.9951 | 3.6176 | 3.7269 | 3.4608 | 3.0611 |
|  | MPWB95 | 4.1440 | 3.7296 | 3.8595 | 3.6112 | 3.2122 |
|  | BMK | 4.4854 | 4.0017 | 4.1855 | 3.9682 | 3.5418 |
|  | M06-2X | 4.5737 | 4.1302 | 4.3403 | 4.1815 | 3.7895 |
|  | M06-HF | 4.9308 | 4.5022 | 4.6173 | 4.8088 | 4.5122 |
| ***E*_VA_ (T_1_)**  **(eV)** | B3LYP | 3.3001 | 3.2539 | 3.3098 | 3.2647 | 2.8620 |
|  | PBE1PBE | 3.2200 | 3.2452 | 3.2345 | 3.2426 | 3.0451 |
|  | MPWB95 | 3.4268 | 3.4527 | 3.4468 | 3.4548 | 3.1986 |
|  | BMK | 3.5617 | 3.6128 | 3.5899 | 3.5965 | 3.5268 |
|  | M06-2X | 3.7310 | 3.7666 | 3.7630 | 3.7687 | 3.7720 |
|  | M06-HF | 4.0122 | 4.0871 | 4.0570 | 4.0608 | 4.0677 |
| **CT amount (*q*)** | | 0.8555 | 0.6579 | 0.8305 | 0.8575 | 0.8536 |
| **Optimal HF%** | | 36 | 28 | 35 | 36 | 36 |
| ***E_VA_* (S_1_, OHF) (eV)** | | 4.28 | 3.68 | 3.99 | 3.78 | 3.38 |
| ***E_0-0_* (^1^CT) (eV)** | | 4.04 | 3.44 | 3.75 | 3.54 | 3.14 |
| ***E_0-0_* (^3^CT) (eV)** | | 3.46 | 3.21 | 3.46 | 3.52 | 3.13 |
| ***E_0-0_* (^3^LE) (eV)** | | 3.07 | 3.05 | 3.03 | 3.10 | 3.04 |

| **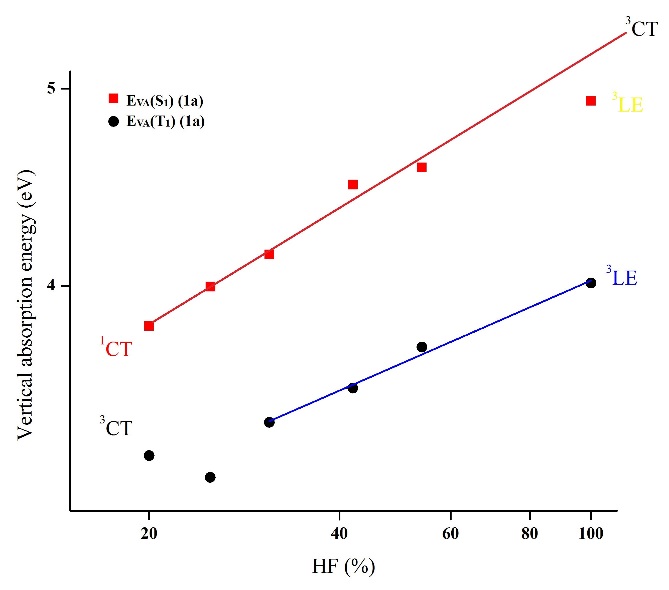** | **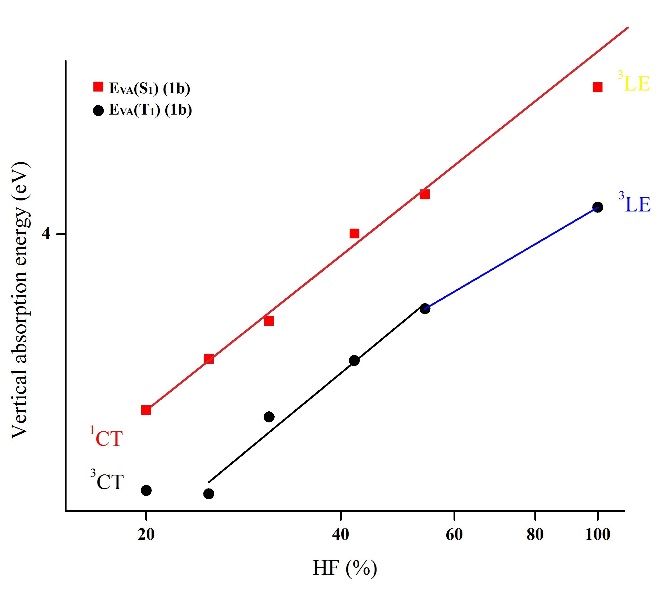** |
| --- | --- |
| **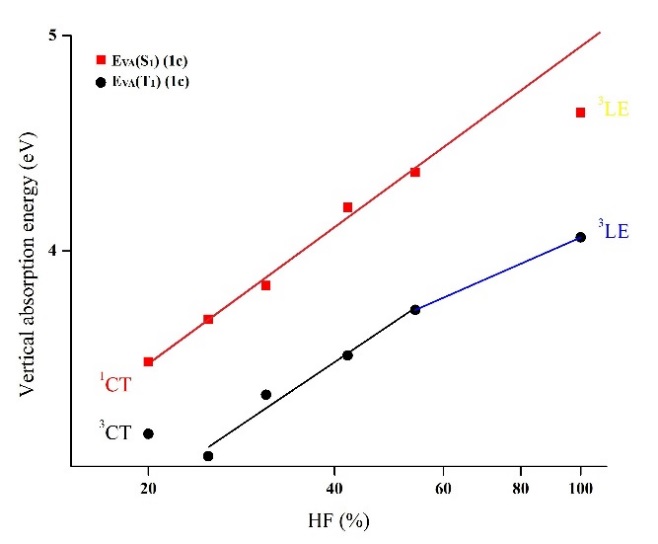** | **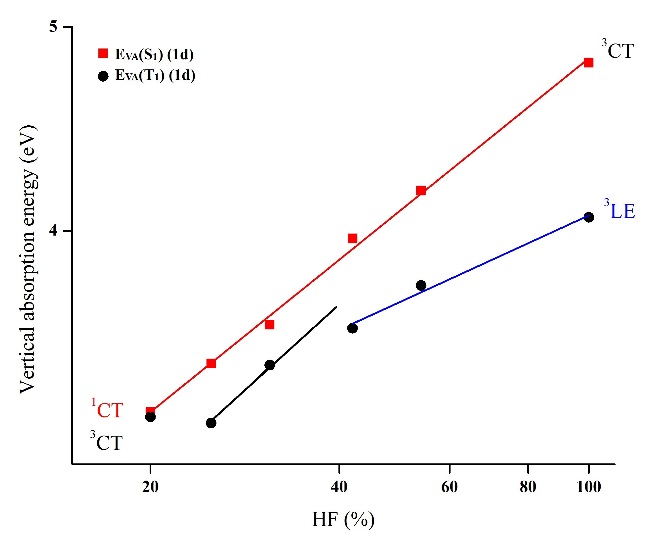** |
| **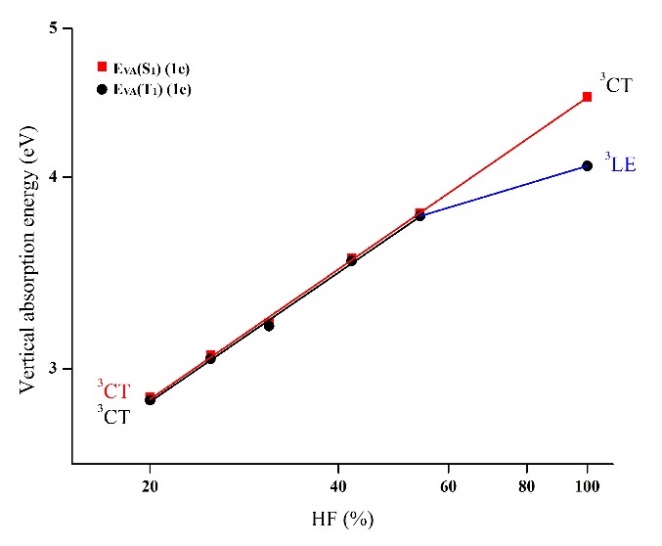** | **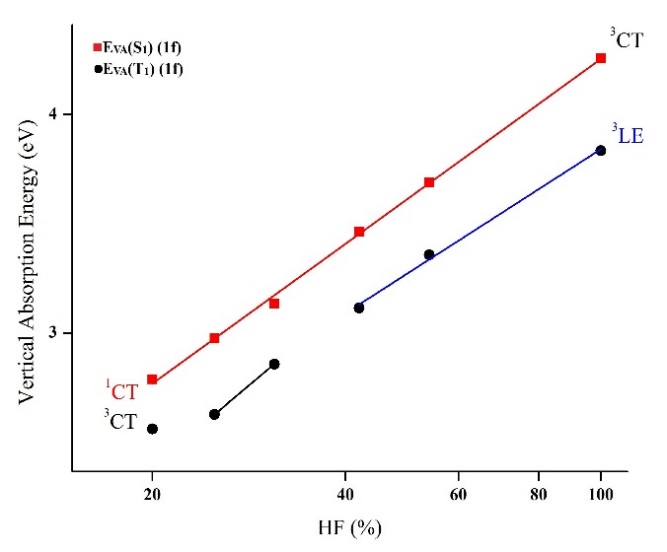** |
| **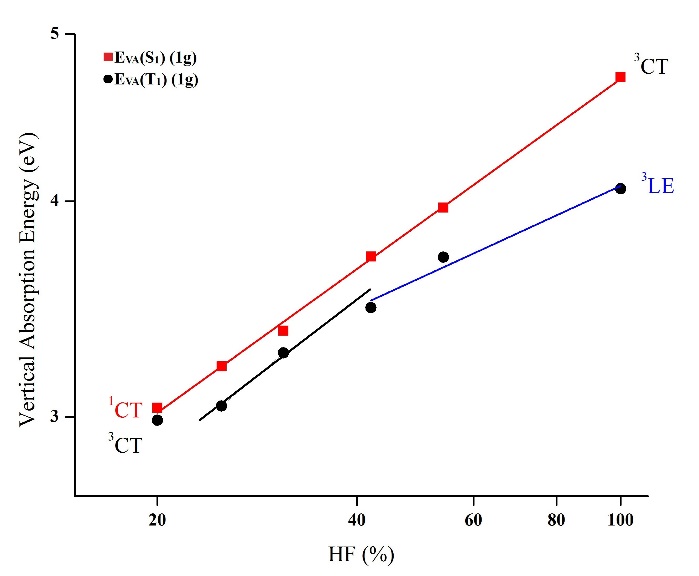** | **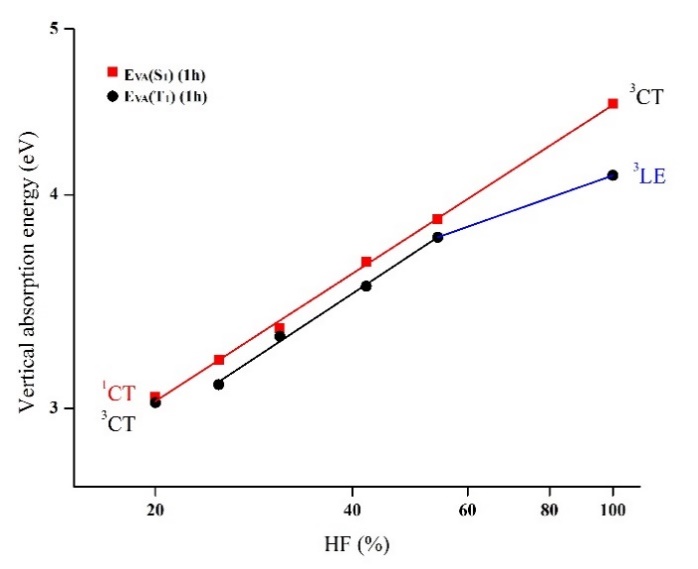** |

# **Figure S2** Dependence of *E*_VA_(S_1_) and *E*_VA_(T_1_) on the HF% in TD-DFT plotted on a log-log scale.
