# Supplementary material for: Exploration of violet-to-blue thermally activated delayed fluorescence emitters based on “CH/N” and “H/CN” substitutions at diphenylsulphone acceptor. A DFT study
Source: Front Chem. 2023 Nov 9;11:1279355. doi: 10.3389/fchem.2023.1279355 (PMC10666053; doi:10.3389/fchem.2023.1279355)

Violet-to-Blue TADF materials with small ΔE_{ST} were designed via "CH/N" and "H/CN" substitution at DPS-unit of DMDHNP-DPS using OHF method.

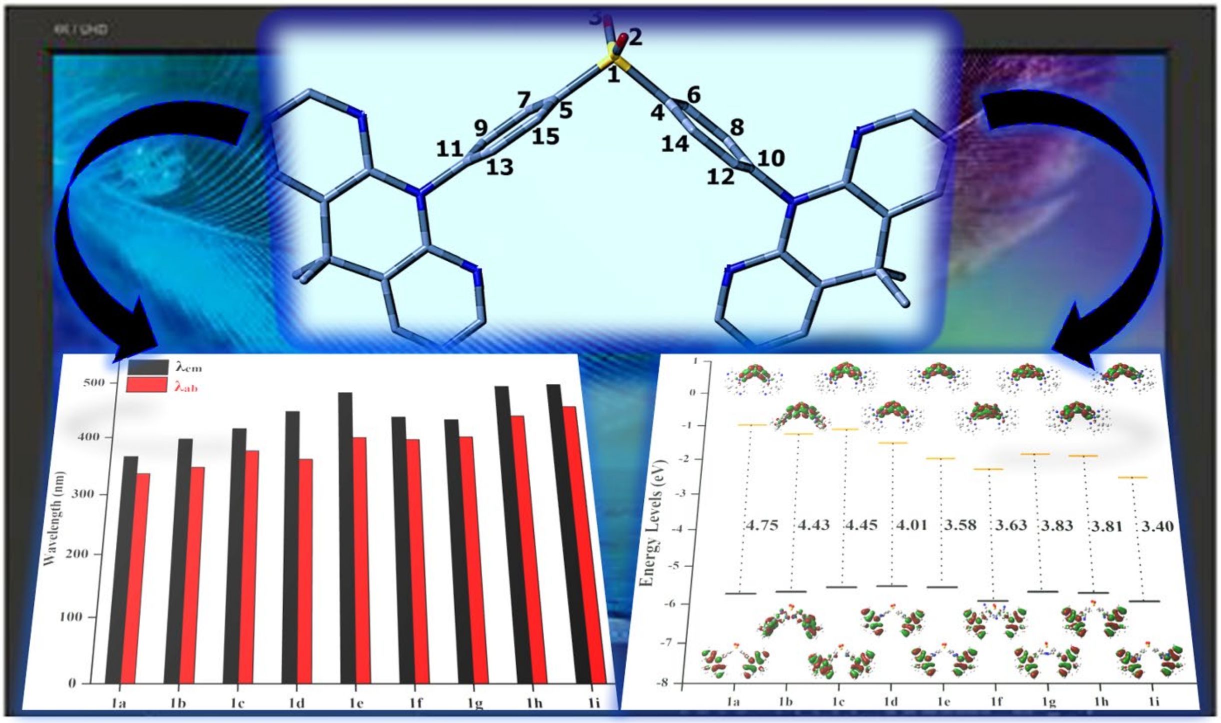

Supplement: Supplementary file 2 [file Image1.pdf]
